# Differential effects of vascular endothelial growth factor on glycocalyx of endothelial and tumor cells and potential targets for tumor metastasis

**DOI:** 10.1063/5.0064381

**Published:** 2022-01-18

**Authors:** Yifan Xia, Yunfei Li, Bingmei M. Fu

**Affiliations:** Department of Biomedical Engineering, The City College of the City University of New York, New York, New York 10031, USA

## Abstract

On the surface of every mammalian cell, there is a matrix-like glycocalyx (GCX) consisting of proteoglycans and glycosaminoglycans (GAGs). Disruption of endothelial cell (EC) GCX by a vascular endothelial growth factor (VEGF, VEGF-A_165_), a tumor secretion, was found to be an early event in tumor cell (TC) metastasis across vascular barriers. However, how the TC secretion VEGF affects its own GCX is unknown. To investigate the VEGF effect on TC GCX and to elucidate the ultrastructural organization of EC and TC GCX and their alteration by VEGF, we employed super-resolution stochastic optical reconstruction microscopy to observe the spatio-chemical organizations of the heparan sulfate (HS) and hyaluronic acid (HA), two representative GAGs of GCX, on human cerebral microvascular endothelial cells (hCMEC) and malignant breast cancer cells MDA-MB-231 (MB231). We found that HS and HA have distinct organizations on hCMEC and MB231. Only HS of hCMEC is perpendicular to the cell surface, while HA of hCMEC as well as HS and HA of MB231 all lie in the same plane as the cell surface where they appear to weave into a 2D network covering the cell. We also found that VEGF significantly reduces the length and coverage of HS on hCMEC but does not change the thickness and coverage of HA on hCMEC. On the contrary, VEGF significantly enhances the coverage of HS and HA on MB231 although it does not alter the thickness. The differential effects of VEGF on the GCX of TC and that of EC may favor TC adhesion and transmigration across EC barriers for their metastasis.

## INTRODUCTION

On the surface of every mammalian cell, there is a matrix-like glycocalyx (GCX) layer of a mucopolysaccharide structure consisting of glycoproteins, acidic oligosaccharides, terminal sialic acids (SA), proteoglycans, and glycosaminoglycans (GAGs), including heparan sulfate (HS), chondroitin sulfate (CS), and hyaluronic acid (HA). The GCX components may be different in different types of cells for their diverse functions. For example, the GCX at the endothelial cells (ECs) lining the inner side of our blood vessels is a mechano-sensor to the blood flow, a regulator controlling the material exchange between circulating blood and the surrounding tissue and a barrier restricting the interaction between the circulating cells and the ECs forming the vascular wall.^1–3^

Although many functions have been found for the EC GCX, the investigation for the functions of tumor cell (TC) GCX has just started in recent years.^4–9^ Recent studies found that a bulkier GCX on TCs is associated with increased migration^10^ and metastatic potential of cancers.^11^ The TC GCX responds to the interstitial flow-induced shear forces by secreting matrix metalloproteinases to degrade the surrounding ECM. This makes it easier for TCs to migrate through the tissue and invade the nearby vasculature.^12–15^ The bulky GCX on the circulating TCs, such as HA, creates not only a barrier to therapeutic agents but also a shield to the blood flow induced friction forces.^16^

Metastasis is a hallmark of cancer. Adhesion to and transmigration across the blood-brain barrier (BBB) are two critical steps in breast cancer hematogenous metastasis to the brain.^17–19^ To investigate the structural and molecular mechanisms by which TCs adhere to and transmigrate across the EC barrier, by directly injecting breast cancer MDA-MB-231 (shorten as MB231) cells into individual microvessels on rat mesentery at physiological flow rates, a prior study showed that after 5–6 min and ∼45 min MB231 cell perfusion, the HS of GCX on the microvessel wall decreases to ∼44% and ∼18% of the control, respectively.^20^ It was also shown that reinforcing GCX of the microvascular wall by applying a plasma protein, orosomucoid, or a plasma sphingolipid, sphingosine-1-phosphate, can significantly reduce the MB231 adhesion.^20–23^ Another study investigating MB231 adhesion to and transmigration across an *in vitro* BBB formed by mouse brain microvascular ECs (bEnd3) demonstrated that 1 h MB231 adhesion to bEnd3 monolayer significantly degrades the HS of GCX to ∼40% of the control.^24^ Prior studies also investigated the effect of vascular endothelial growth factor (VEGF, VEGF-A_165_), a tumor secretion for angiogenesis and microvascular hyperpermeability,^25^ on MB231 adhesion to the wall of individual post-capillary venules on rat mesentery and found that 1 h pretreatment with 1 nM VEGF significantly increases the microvessel permeability and enhances the TC adhesion.^26^ In consistent with the *in vivo* study, an *in vitro* study also found that VEGF significantly increases bEnd3 monolayer permeability and MB231 adhesion to and transmigration across this *in vitro* BBB.^24^ The increased EC permeability by VEGF is due to the degradation of EC GCX and disruption of junction proteins.^24,27–29^

The above-described prior studies have shown that TC adhesion degrades EC GCX by a TC secretion VEGF and other factors, and degradation of EC GCX increases TC adhesion and transmigration. However, how the TC secretion VEGF affects its own GCX to enhance its metastatic ability is unknown. Therefore, one objective of this study was to test the hypothesis that VEGF can reinforce the GCX of TCs because prior studies have reported that a bulkier GCX increases migration^10^ and metastatic potential of cancers.^11^ Another objective was to test the hypothesis that the GCX on ECs and that on TCs have distinct ultra-structure and organization. To test these hypotheses, we employed super-resolution stochastic optical reconstruction microscopy (STORM) from Nikon (N-STORM) to observe the HS and HA, two representative GAGs of GCX, on human cerebral microvascular endothelial cells (hCMEC) and malignant breast cancer cells MDA-MB-231 (MB231).

The recently developed STORM, one type of single molecule localization microscopy,^30^ employs organic dyes and fluorescent proteins as photo-switchable emitters to trade temporal resolution for a superspatial resolution. Using fluorescently conjugated antibodies to label the GCX components, the N-STORM in the current study offered us a ∼20 nm lateral and ∼50 nm axial resolution, which is much higher than that of conventional confocal microscopy, ∼200–300 nm lateral and ∼500–600 nm axial resolution for a high magnification objective and numerical aperture lens (e.g., 63×/NA1.4) with the commonly used laser of wavelength 500–700 nm, due to the light diffraction limit.^31,32^ In Appendix A, we detailed the working principle of STORM and explained why it can overcome the light diffraction barrier to provide a super-resolution at a nanometer scale. By using STORM, we avoided not only the dehydration artifact in electron microscopy but also the limitation of the spatial resolution in conventional fluorescence microscopy. With data processed by the affiliated algorithm in the N-STORM system,^33,34^ we revealed, for the first time, the super-resolution images of HS and HA elements of the GCX at the hCMEC monolayer and at the surface of MB231. We also determined the ultra-structural parameters of HS and HA under control and after VEGF treatment.

## RESULTS

### Differential effects of VEGF on glycocalyx of hCMEC and MB231 observed by confocal microscopy

**FIG. 1.**
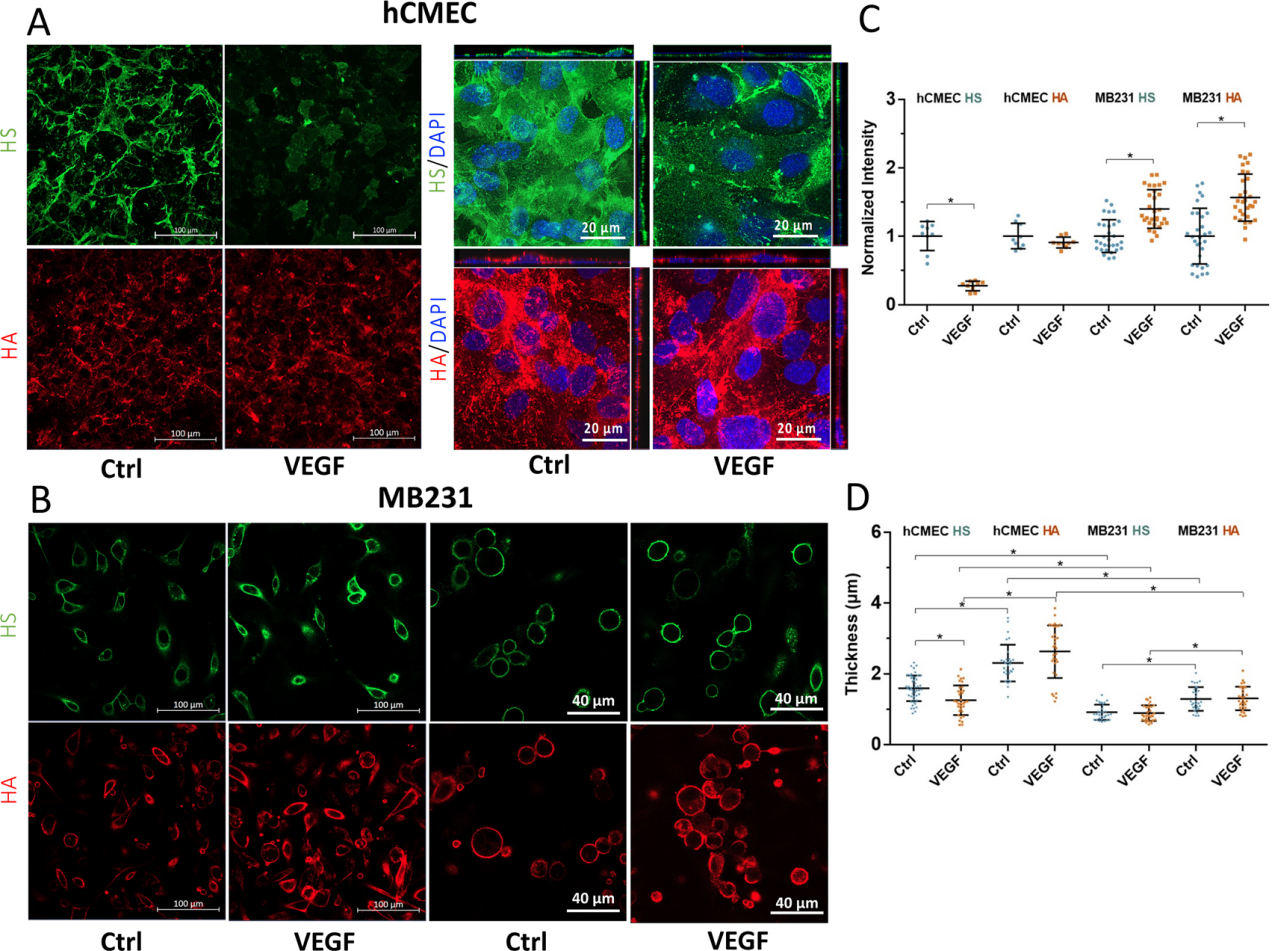
Effects of VEGF on glycocalyx of hCMEC and MB231 observed by confocal microscopy. Confocal images of (a) HS and HA of GCX on hCMEC and (b) HS and HA of GCX on MB231 under control and after VEGF treatment. The first two columns in (a) and (b) are the images collected by a 40×/NA1.3 lens. The scale bar is 100 *μ*m. The third and fourth columns are images collected by a 63×/NA1.4 lens. (c) Comparison of HS and HA intensity under control and after VEGF treatment for hCMEC and MB231. Normalized HS and HA intensity for the control and after VEGF treatment. n = 3 samples with 3 fields in each sample (each field 320 × 320 *μ*m) analyzed for each case for the HS and HA at hCMEC monolayer. n = 3 samples with 10 cells in each sample analyzed for each case for the HS and HA at MB231 from the images shown in the first two columns in (b). (d) Thickness of HS and HA at HCMEC and MB231 under control and after VEGF treatment. n = 3 samples with 10 cells in each sample analyzed for each case for both hCMEC and MB231 from the images shown in the third and fourth columns of (a) and (b). Plots are mean ± SD. ^*^ p < 0.05.

After hCMECs reached confluency (Fig. 7 in Appendix B), MB231 cells were adherent and some cells spread out but were not confluent on the glass-bottom dish, the cells were fixed and immunostained for the GCX (see METHODS below). To have an overview on the effects of VEGF on GCX of hCMEC and MB231 cells, we first employed confocal microscopy to observe the heparan sulfate (HS) and hyaluronic acid (HA) of GCX under control and after 2 h treatment with 1 nM VEGF (VEGF A_165_, Peprotech, Rocky Hill, NJ). Figures 1(a) and 1(b), demonstrate the confocal images of HS (green) and HA (red) on hCMEC and MB231 under control and after VEGF treatment. The first two columns in Figs. 1(a) and 1(b) are the images collected by a 40×/NA1.3 lens, which were used to quantify the overall intensity changes in HS and HA by VEGF. Figure 1(c) compares the intensity changes in HS and HA by VEGF and shows that although VEGF significantly reduces the HS to 26.3% (p < 0.02) and insignificantly reduces HA to 89.7% (p = 0.40) of their respective controls on hCMECs, surprisingly, VEGF greatly increases HS to 138.5% (p < 0.01) and HA to 155.8% (p < 0.02) of their respective controls on MB231. The third and fourth columns in Figs. 1(a) and 1(b) are the images collected by a 63×/NA1.4 lens, which were used to estimate the thickness of HS and HA under each case. The method for determining the thickness is described in Fig. 8 in Appendix C. Figure 1(d) shows the thickness changes in the HS and HA by VEGF. VEGF only significantly decreases the thickness of HS at hCMEC from 1.56 ± 0.32 to 1.29 ± 0.43 *μ*m (p < 0.01). It does not have significant effects on the thickness of HA at hCMEC and those of HS and HA at MB231. Figure 1(d) also shows that the HA seems to have a larger thickness compared to that of the HS in both hCMEC and MB231.

### Differential organization of glycocalyx components on hCMEC and MB231

**FIG. 2.**
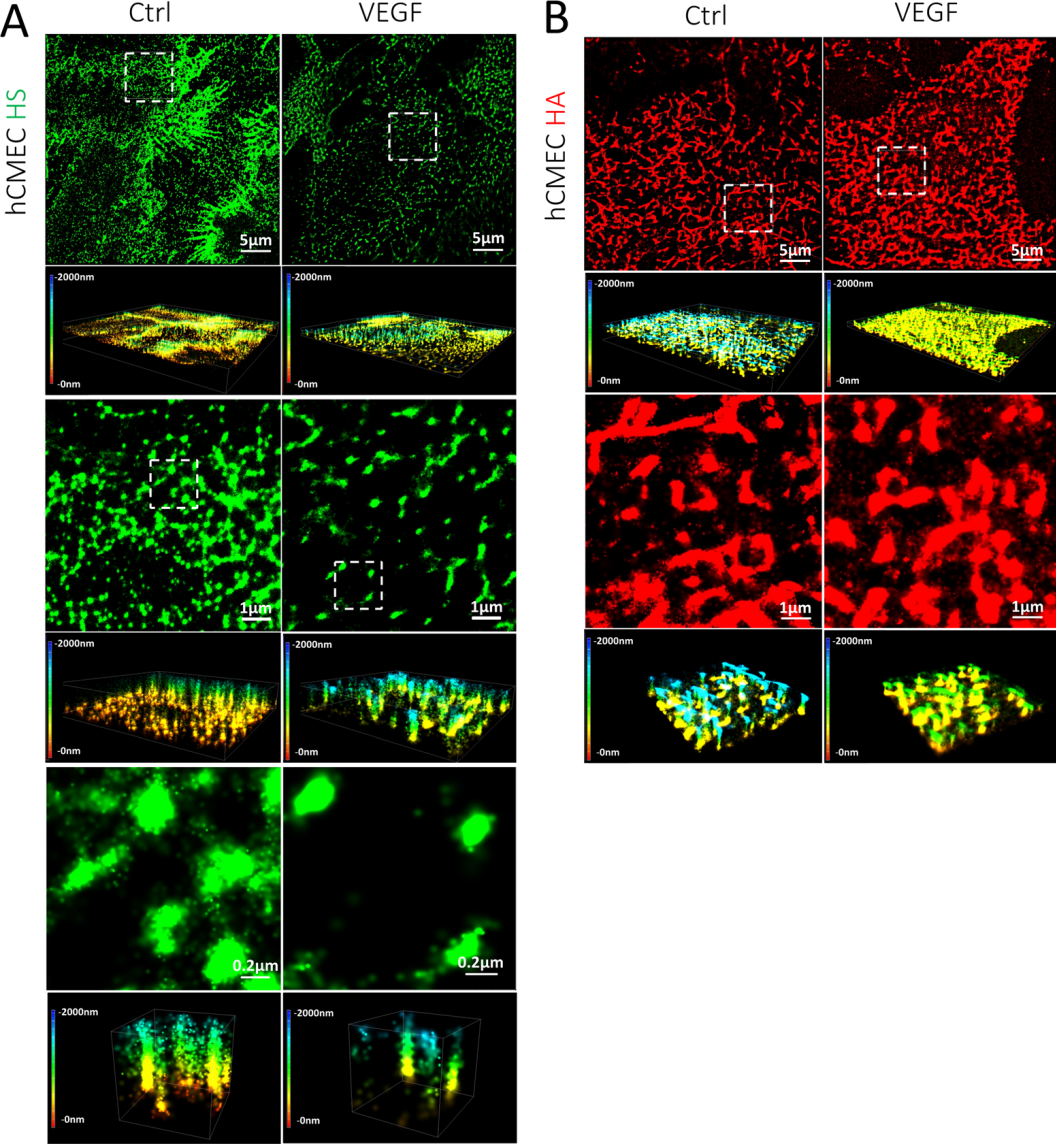
STORM images of HS and HA elements on the apical surface of hCMEC. (a) HS and (b) HA under control (left column) and after VEGF treatment (right column). The first row shows the top views (projection or 2D) of the 3D images of 40 × 40 *μ*m (second rows) for the HS or HA on the cell surface. The third row shows the top views of the regions enclosed by the white dashed line (8 × 8 *μ*m) in the first row, and the fourth row shows the corresponding 3D views. The fifth row in (a) shows the top views of the regions enclosed by the white dashed line (1.6 × 1.6 *μ*m) in the third row. The scale bar for the images in the fifth row is 0.2 *μ*m. The sixth row shows the corresponding 3D views of the images in the fifth row. The color bar at the left of the 3D images is the depth (length) scale, from 0 to 2000 nm. 0 is located at the cell apical surface. The quantification method for the length, diameter, and thickness of HS and HA elements is described in Appendix D and in Data analysis of Methods section.

**FIG. 3.**
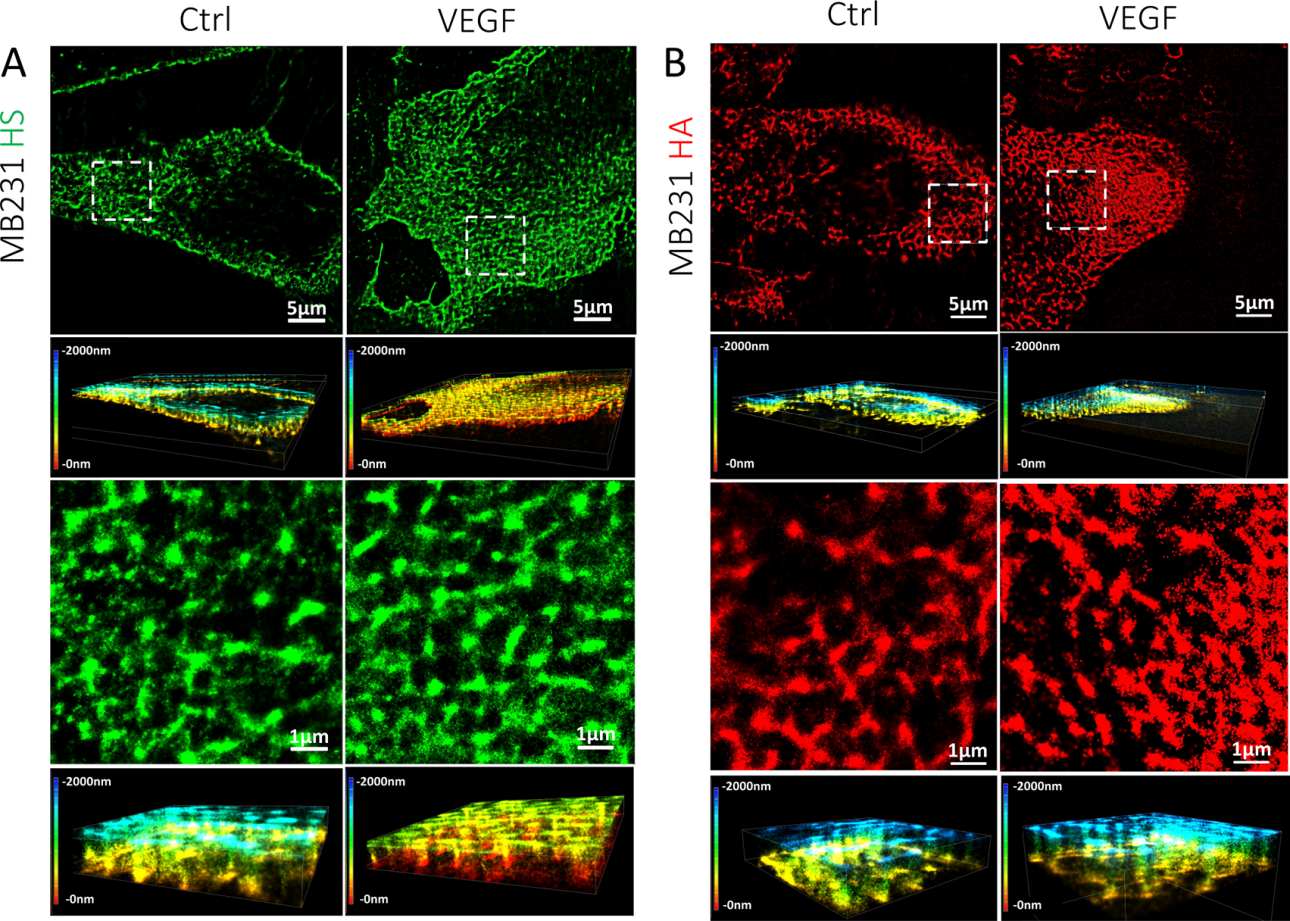
STORM images of HS and HA elements on the surface of MB231. (a) HS and (b) HA under control (left column) and after VEGF treatment (right column). The first row shows the top views (projection or 2D) of the 3D images of 40 × 40 *μ*m (second rows) for the HS or HA on the cell surface. The third row shows the top views of the regions enclosed by the white dashed line (8 × 8 *μ*m) in the first row, and the fourth row shows the corresponding 3D views. The color bar at the left of the 3D view represents the depth scale, from 0 to 2000 nm. 0 is located at the cell surface. The quantification method for the diameter and thickness of HS and HA elements is described in Appendix D and in Data analysis of Methods section.

As explained in the introduction and Appendix A, the currently used confocal microscopy can only reach ∼200–300 nm lateral and ∼500–600 nm axial resolution due to the light diffraction limit. The confocal images shown in Figs. 1(a) and 1(b) are not able to provide us the ultrastructure and organization of GCX. We, thus, used super-resolution N-STORM for the visualization of GCX. A set of data movies of blinking dots in a region of 40 × 40 *μ*m were first collected by the STORM. The single-emitter centroid algorithm was applied to estimate the 3D locations of activated fluorophores in the data movie and produced a 3D STORM image with the spatial resolutions of ∼20 nm in the lateral plane (projection of 3D view, or top view or 2D view plane) and ∼50 nm in the axial direction (vertical direction) as shown in Figs. 2 and 3.

Figures 2 and 3 demonstrate the organization of HS (green) and HA (red) of GCX revealed by STORM on hCMEC and MB231, respectively. Figures 2(a) and 2(b) show the distribution of HS and that of HA observed at the luminal (apical) surface of hCMEC. Figure 2(a) shows that HS elements appear to be perpendicular (or tilted) to the luminal surface of hCMEC (the fourth and the sixth rows), while Fig. 2(b) shows that HA elements lie in the same plane as the cell surface (the fourth row). HA elements appear to interweave into a 2D network covering the hCMEC surface. Figures 3(a) and 3(b) show the organization of HS and that of HA observed at the surface of MB231. In contrast to the organization of HS elements at hCMEC, HS elements at MB231 appear to lie in the same plane as the cell surface. Both HS and HA elements seem to form a 2D network sheet covering the MB231 cell surface.

### Ultrastructural parameters of HS and HA elements on hCMEC and MB231 under control

**FIG. 4.**
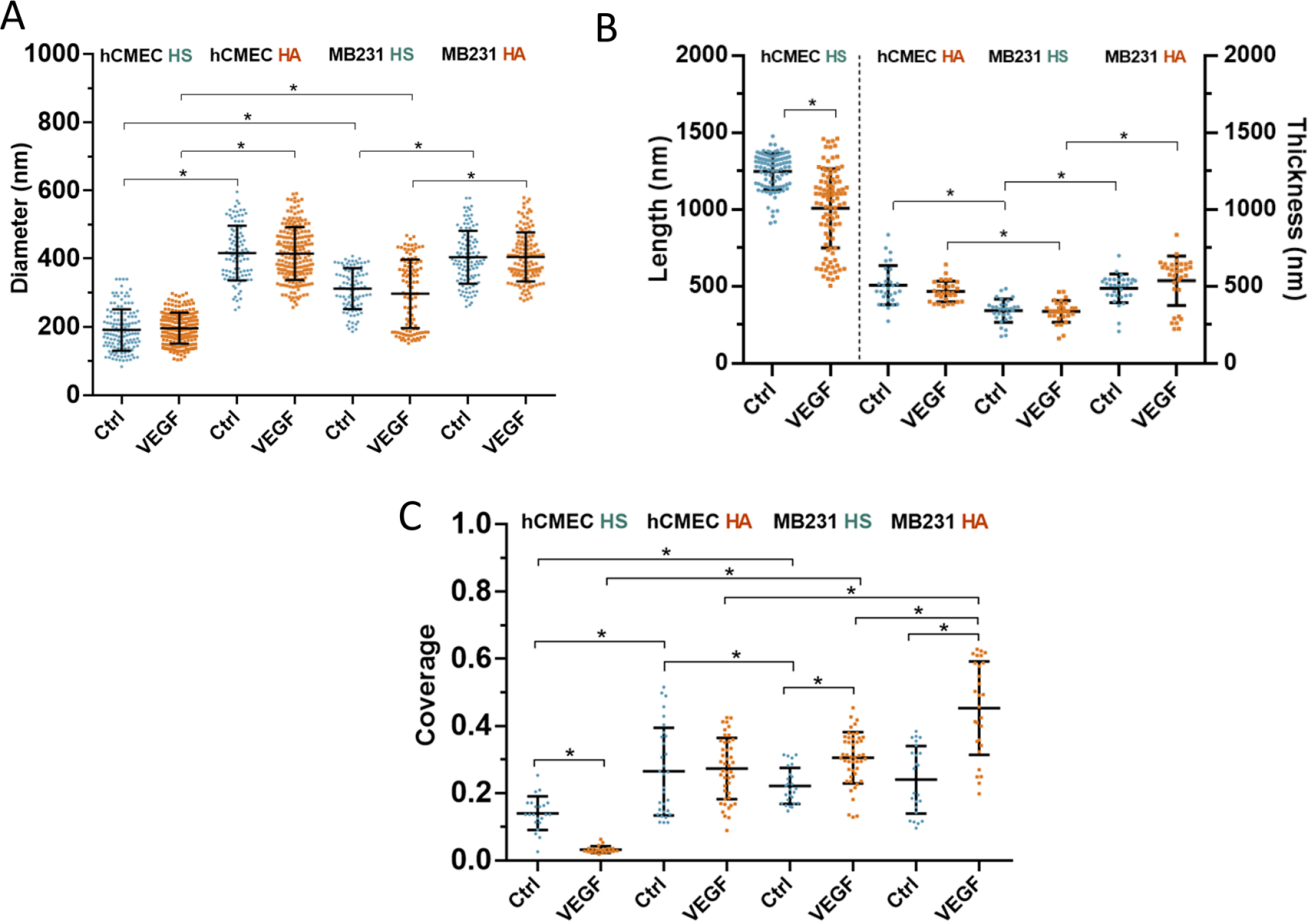
Quantification of HS and HA elements at hCMEC and MB231 under control and after VEGF treatment. (a) Diameter of HS and HA at hCMEC and at MB231. (b) Length of HS at hCMEC, thickness of HA at hCMEC, and thickness of HS and HA at MB231. The vertical dotted line separates the length of individual HS elements at hCMEC and the thicknesses of HA at hCMEC, and HS and HA at MB231. (c) Coverage of HS and HA at hCMEC and at MB231. The quantification method for the diameter, length, thickness, and coverage of HS and HA elements is described in Appendix D and in Data analysis of Methods section. Plots are mean ± SD. ^*^ p < 0.05.

The NIS-Elements software in the N-STORM system was employed to estimate the ultrastructural parameters of HS and HA from the 3D images shown in Figs. 2 and 3. The details for how to quantify the length, thickness, diameter, and coverage of GCX elements were described in the METHODS section and in Appendix D. The values of these parameters are summarized in Fig. 4. We should realize that the estimated parameters of the HS or HA are for the fluorescent conjugates, e.g., antibodies, used to visualize these elements, not the parameters for a single molecule. In fact, a single HA molecule has a diameter, which is less than 1 nm.^35^ The diameter of the observed HS element at hCMEC is 191± 69 nm (n = 139) and that of HS at MB231 is 306 ± 62 nm (n = 112). The diameter of HS at MB231 is significant bigger than that at hCMEC (p < 0.01). However, the diameter of HA at MB231, 404 ± 78 nm (n = 113), is comparable to that of HA at hCMEC, which is 416 ± 80 nm (n = 107) (p = 0.26). These results suggest that HA elements of hCMEC and MB231 are from the same core protein, but HS elements might be from different core proteins.

Since HS elements of hCMEC appear to be perpendicular to the cell apical surface, the length of HS at hCMEC measured from the cell surface is 1250 ± 118 nm (n = 106). While HA of hCMEC and HS and HA of MB231 seem to lie in the same plane as the cell surface, the thickness of HA at hCMEC is 508 ± 127 nm (from n = 30 regions), the thickness of HS at MB231 is 342 ± 77 nm (from n = 30 regions) and that of HA at MB231 is 487 ± 94 nm (from n = 30 regions). Apparently, there is no difference between the thickness of HA at hCMEC and that of HA at MB231 (p = 0.46). However, the thickness of HA at MB231 is larger than that of HS at MB231 (p < 0.01).

For the HS distribution at hCMEC, the density is 4.8 ± 1.7 elements/*μ*m^2^ measured from the images in Fig. 2(a). This density was converted to the coverage by density x π(D/2)^2^ = 13.7 ± 4.9% (D is the average diameter of HS = 191 nm). For the HS distribution on MB231, the coverage is 22.1 ± 5.4%. For the HA distribution, the coverage is 26.4 ± 13.0% and 24.0 ± 10.0%, respectively, at hCMEC and MB231. Although HS elements cover more at MB231 than at hCMEC (p < 0.01), no difference in the coverage of HA between these two types of cells (p = 0.42).

### Differential effects of VEGF on glycocalyx of hCMEC and MB231 revealed by STORM

After VEGF treatment, Fig. 4(a) shows that diameter of HS at hCMEC becomes 193 ± 45 nm (n = 200) and that of HS at MB231 becomes 297 ± 101 nm (n = 111), the diameter of HA at hCMEC becomes 415 ± 78 nm (n = 197) and that of HA at MB231 becomes 405 ± 72 nm (n = 128), respectively. Compared to their diameters under control, there is no change in the diameters of HS and HA on both hCMEC and MB231 after VEGF treatment, suggesting that VEGF does not change the ultrastructure of individual GAG components. However, Fig. 4(b) shows that VEGF significantly reduces the length of HS at hCMEC to 1032 ± 284 nm (n = 100) (p < 0.01), 82.6% of its control value but VEGF does not alter the thickness of HA at hCMEC, neither the thickness of HS nor HA at MB231. Figure 4(c) demonstrates that VEGF greatly decreases the coverage of HS at hCMEC from 13.7± 4.9% to 3.2± 1.1% (p < 0.01). It also shows that VEGF does not change the coverage of HA at hCMEC; however, it significantly increases the coverage of HS at MB231 from 22.1 ± 5.4% to 30.5 ± 7.6%, 1.38-fold (p < 0.01), and that of HA at MB231 from 24.0 ± 10.0% to 45.3 ± 13.9%, 1.89-fold (p < 0.01). The results shown in Fig. 4 indicate that VEGF has differential effects on the GCX of hCMEC and that of MB231.

## DISCUSSION

Using the super-resolution N-STORM system, we revealed the spatio-chemical organization of HS and HA in the GCX of hCMEC and MB231 at the nanometer scale. Our results show that HS elements are perpendicular to while HA elements lie in the same plane as the luminal surface of hCMEC and form a net-like coat. However, for MB231, both HS and HA elements lie in the same plane at the cell surface, also form a web-like cover. We found that the tumor secretion, VGEF-A_165_, does not alter the diameter of HS and HA at hCMEC nor at MB231. While VEGF greatly reduces the length and coverage of HS at hCMEC, it does not reduce the thickness and coverage of HA at hCMEC. In contrast, VEGF significantly increases the coverage of HS and HA at MB231 although it does not alter their thickness.

The spatial organizations of HS and HA on hCMEC (human cerebral microvascular endothelial cells) are the same as those observed on bEnd3 (mouse brain microvascular endothelial cells) by STORM in Ref. 33 in which they showed shorter (∼600 nm length) HS elements and smaller HA elements (∼160 nm diameter), indicating that the GCX components on ECs serve the same functions, such as a mechano-sensor to the blood flow and a barrier to material exchange and to the interaction between circulating cells and ECs lining the blood vessel wall.^1–3^ The perpendicular and taller HS elements (1250 nm in length and 191 nm in diameter) at hCMEC would favor them as a mechano-sensor to the blood flow, like primary cilia at human microvascular ECs with ∼200 nm diameter and 1.1–16.5 *μ*m length,^36–38^ although HS elements have much larger coverage, 13.8% compared to that of primary cilia, ∼0.05%. We also observed cable-like HS structures at hCMEC with an average length of 3.89 *μ*m (ranges from 2.48 to 6.66 *μ*m) under control but not after VEGF treatment [the first row in Fig. 2(a)]. A cable-like HA structure was reported on human lung microvascular ECs after treatment with inflammatory stimuli but not under control.^39,40^ It was said that inflammatory stimuli increase HA synthesis to form a cable-like adhesive structure for mediating leukocyte adhesion.^40^ What the exact function of cable-like HS structures is at hCMEC is unclear, but most likely, HS elements could serve as a mechanosensor to the blood-flow induced tangential force (drag) and torque,^41^ and as a barrier limiting the interaction between circulating cells including TCs and ECs forming the vessel wall as well as regulating the transvascular material exchange.

The tumor secretion, VEGF, not only reduces the length but also the coverage of HS elements at hCMEC, resulting in a less sensitive mechano-sensor and a compromised HS barrier, which increases the microvascular permeability and the adhesion of circulating TCs to the ECs. The VEGF-induced decrease in the HS length and coverage on hCMEC revealed by STORM is consistent with the prior observation that the intensity of HS at ECs was reduced after VEGF treatment.^20,24^ Their studies also found that the permeability of EC barriers and TC adhesion/transmigration are increased by VEGF along with the decreased HS at ECs. The mechanisms by which VEGF reduces HS of ECs are unclear. The likely one is via VEGF receptor 2 (VEGFR2), KDR/Flk-1, since inhibiting KDR/Flk-1 by SU-1498 can abolish the increased EC permeability and TC adhesion by VEGF *in vitro* and *in vivo*.^26,42^ VEGFR2 signaling was found to contribute to the glycosaminoglycan regulation by VEGFA in endothelial glycocalyx.

On the other hand, larger sized HA elements (416 nm diameter) at hCMEC form a web-like structure coating the hCMEC surface with a thickness of 508 nm and a coverage of 22.1%. The organization and structure of HA elements would favor them as a barrier to the transvascular transport of water and solutes (molecular sieve) and a barrier for the interaction of circulating cells and ECs.^39^ They could also serve as a mechano-sensor to the shear stress at the EC surface.^43^ Interestingly, VEGF does not alter the size, neither the thickness nor the coverage of HA elements at hCMEC. Unchanged HA of ECs by VEGF might assist TC adhesion like inflammatory stimuli-enhanced HA at ECs mediate leukocyte adhesion.^40^ However, different from inflammatory stimuli-induced cable-like HA structures at human lung microvascular ECs, no cable-like structures were found for HA elements after VEGF treatment, neither under the control.

On the contrary, at MB231, HS elements are not perpendicular to the cell surface; instead, they are in the same plane as the cell surface, the same as HA elements. The diameter of HA, 401 nm is bigger than that of HS, 306 nm. The thickness of HA elements, 487 nm, is also larger than that of HS, 342 nm. The 2D network sheet formed by both HS and HA covers the surface of MB231. It can provide a shield for the circulating TCs from being damaged by the blood-flow induced shear forces.^16^ VEGF does not alter the size and thickness of HS and HA at MB231; surprisingly, it significantly increases the coverage of both HS and HA on MB231 by 1.36-fold and 1.89-fold, respectively, just opposite to its effect on HS of hCMEC. The reasons for the enhanced GCX on MB231 by VEGF are unknown. One possibility is that VEGF may stimulate the HS and HA synthesis as inflammatory stimuli increase the HA synthesis.^39,40^ Another possibility is to protect TCs from physical damage and therapeutic treatments, as well as immune surveillance.^16,44^ The third possibility is that the VEGF-enriched HA on MB231 can help TCs adhere to the microvessel wall since Offeddu *et al.*^7^ reported that firm adhesion of TCs to the endothelium is through accumulation of deposited HA in the microvasculature, which could prime a niche for subsequent TCs to adhere through CD44 binding. The accumulated HA may come from the shedding of the enriched HA of TCs by VEGF and from the unaltered HA of ECs by VEGF. HA is a non-sulfated GAG, which binds with its surface receptors CD44 and receptor for HA-mediated motility (RHAMM).^45^ Further studies are necessary to test these hypotheses.

Our finding for the differential size and organization of HS elements at hCMEC (smaller diameter and perpendicular to the cell surface) and those at MB231 (larger diameter and lie in the same plane as the cell surface) suggests that they may be from different core proteins. The core proteins for the HS at ECs are syndecans, glypicans, and perlecan.^46^ Syndecans and glypicans are also found at TCs.^47,48^ To confirm if the HS at ECs and that at TCs have different core proteins, in the future study, we will either co-label the core protein (e.g., syndecan-1 and glypican-1) with HS at ECs and TCs or use shRNA to knockdown/knockout specific core proteins before observing the HS elements on these two types of cells.

Conventional EM has been used to observe the GCX thickness at blood vessels since 1960s.^49^ The reported thickness ranges from less than 100 nm to 0.5 *μ*m because of dehydration artifacts associated with aqueous fixatives that likely dissolve all but the protein cores of proteoglycans and collapse the inherently hydrated structures.^50–53^ By employing laser scanning confocal microscopy and multi-photon microscopy, and fluorescently tagged antibodies to HS or HA binding protein, or wheat germ agglutinin (WGA) to label SA residues of the GCX, the thickness of GCX at blood vessels was found to be ∼0.5–4.5 *μ*m for different types of vessels, from microvessels to aortas.^54–57^ In our current study, the thickness of HS elements at hCMEC observed by confocal microscopy is 1.53 *μ*m and the thickness of HA is 2.23 *μ*m, comparable to those observed at the microvessels.^56,57^ The length of HS and the thickness of HA at hCMEC revealed by super-resolution STORM (20 nm lateral and 50 nm axial) are 1250 nm (1.25 *μ*m) and 508 nm (0.51 *μ*m), respectively. These values are smaller than those estimated from the confocal images (see Fig. 8) due to the much poorer resolution of confocal microscopy (200–300 nm lateral and 500–600 nm axial). Ebong *et al.*^58^ presented cryo-EM images of *in vitro* GCX that avoided the dehydration artifacts of conventional EM and observed GCX with thickness ∼5 *μ*m on cultured cell monolayer of rat fat pad ECs (RFPEC). Their value is much larger than what we observed at the hCMEC monolayer by STORM. Although the reason for the discrepancy is unclear, one possibility is that they might observe the cable-like HS structure (∼4 *μ*m at hCMEC, Fig. 2(a) control) in their cryo-EM since for the same RFPEC monolayer, they observed 2.00 *μ*m for HS thickness and 1.85 *μ*m for HA thickness by confocal microscopy, comparable to our measurements at the hCMEC monolayer.

Twamley *et al.*^59^ observed a ∼6.6 *μ*m thick GCX layer at human monocytic THP-1 (leukemia cell line) by EM with a combined high pressure freezing, osmium-free freeze substitution, rehydration, and pre-embedding immunogold labeling method. They also used confocal microscopy to observe the WGA-labeled GCX at THP-1, whose thickness is ∼1 *μ*m. Their observed GCX thickness at THP-1 is comparable to our observed thickness, 0.91 *μ*m for HS and 1.29 *μ*m for HA at MB231 by confocal microscopy. The much larger thickness of GCX observed by EM in Ref. 61 might be also due to the cable-like HA structure at TCs.^60^ The thickness of HS and HA at MB231 observed by STORM is 342 and 487 nm, respectively, which is smaller than that observed by confocal microscopy. Using a super-resolution optical microscopy with 10–20 nm precision in 2D and 3D, Möckl *et al.*^34^ reported that GCX at BT-20 (a breast cancer cell line) is 150–300 nm thick by labeling sialic acids (SA) of GCX. This value is smaller than ours probably because they used smaller dye labeling different GCX components, and their spatial resolution (10–20 nm) is also better than ours (20–50 nm).

Another finding from the current study is that the VEGF-induced GCX changes observed by confocal microscopy are very similar to those revealed by STORM. The reduced amount of HS by VEGF on hCMEC is ∼74% in the intensity observed by the confocal, and ∼81% in the combined length and coverage by STORM. The increased fold of HS and HA by VEGF on MB231 is ∼1.4-fold and ∼1.6-fold in the intensity by confocal microscopy, and ∼1.4-fold and ∼1.9-fold in the coverage by STORM, respectively. However, the STORM images demonstrate more details of differential VEGF effects on the GCX components at hCMEC and MB231 in terms of dimensions and organization. One needs to know that the thickness of the GCX measured by the full width at half maximum (FWHM) (Fig. 8) is not the real thickness because of the inadequate resolution of confocal microscopy. The larger thickness of the GCX (HS and HA) at hCMEC compared to that at MB231 [Fig. 1(d)] is due to the poorer resolution in the z-direction than that at the x-y plane.

As reported in prior studies,^4–9^ there are other GCX components in TCs and Ecs, including mucins, CD44, sialic acid, glycoproteins, such as P-selectin glycoprotein ligand-1 (PSGL-1), and GAGs, such as chondroitin sulfate (CS) and keratin sulfate (KS). Further studies on the effects of VEGF on these GCX components are necessary to fully understand the role of VEGF in EC and TC GCX to facilitate tumor metastasis. In addition, flow also modulates the GCX, we will construct a more biomimetic microchannel with the flow to investigate the effect of VEGF on GCX of the endothelial and tumor cells in the future study.

In summary, the spatio-chemical organizations of the GCX at the surface of EC (hCMEC) monolayer and TCs (MB231) under control and after VEGF treatment were revealed, for the first time, by employing the super-resolution STORM. The ultra-structural parameters of HS and HA were obtained from the reconstructed images and compared for the control and after VEGF treatment. We found that VEGF has opposite effects on the GCX of TCs and that of ECs. The differential effects of VEGF on the GCX of TC and that of EC may favor TC adhesion and transmigration across EC barriers for their metastasis. This finding may facilitate mechanistic understanding and potential therapeutic intervention targeting the glycocalyx-mediated breast cancer brain metastasis. For example, we may use anti-VEGF molecules to neutralize VEGF in the solution, or block VEGF receptors at the ECs and TCs, or use agents to protect/restore GCX at ECs and disrupt GCX at TCs.

## METHODS

### Cell culture

Human brain microvascular endothelial cells (hCMEC/D3 or hCMEC) from Millipore Sigma (Burlington, MA) (passage 7 to 15 after purchase) were cultured in EBM-2 MV endothelial cell growth basal Medium (Lonza, Basel, Switzerland), supplemented with 100 U/ml Penicillin-Streptomycin (Gibco, ThermoFisher, Waltham, MA). Human breast carcinoma cells (MDA-MB-231 or MB231) from ATCC (Manassas, VA) (passage 8 to 18 after purchase) were cultured in Dulbecco's Modified Eagle's Medium (DMEM), supplemented with 10% fetal bovine serum, 100 U/ml penicillin and 100 mg/ml streptomycin sulfate, all from Sigma-Aldrich. Both cells were incubated in the humidified atmosphere with 5% CO_2_ at 37 °C.^24^

### Immunofluorescence labeling of HS and HA

We followed the protocol for immunolabeling HS and HA in Refs. 33 and 63. The hCMECs and MB231 cells were seeded at a density of 60 k/cm^2^ and 20 k/cm^2^, respectively, on the 50 *μ*g/ml collagen I or 30 *μ*g/ml fibronectin coated No.1.5 glass-bottom dish (MetTek, Ashland, MA). After culturing for 4–5 days for hCMECs to reach confluent (Fig. 7) and 2 days for MB231 (adherent and some cells spreading out but not confluent) on the glass-bottom dish, the cells were first gently washed with 1% BSA/PBS for 3 times. Then, they were fixed with 2% paraformaldehyde/0.1% glutaraldehyde^33,61,62^ for 20 min, followed with 0.1% NaBH4 for 7 min. After fixation, they were again washed 3 times with 1%BSA/PBS and blocked with 2% normal goat serum (NGS) for 30 min. For HS labeling, the cells were incubated with mouse anti-heparan sulfate 10e4 (1:100, Amsbio, Cambridge, MA) at 4 °C overnight, followed by an Alexa Fluor 647 conjugated goat anti-mouse IgG (1:100, Sigma-Aldrich, St. Louis, MO); for HA labeling, the cells were incubated with biotinylated hyaluronic acid binding protein (50 *μ*g/ml, Amsbio, Cambridge, MA) at 4 °C overnight, followed by an Alexa Fluor 647 conjugated anti-biotin (1:200, Jackson ImmunoResearch, West Grove, PA). Finally, the samples were post-fixed with 2% paraformaldehyde/0.1% glutaraldehyde for 10 min and then kept in 1% BSA/PBS.^33^ For the sample prepared for the confocal microscopy, we used an Alexa Fluor 488 conjugated goat anti-mouse IgG for HS and labeled cell nuclei with DAPI (SouthernBiotech, Birmingham, AL) in the experiment using 63×/NA1.4 lens.

### Confocal microscopy and quantification of glycocalyx

The samples were imaged by Zeiss LSM 800 confocal laser scanning microscope with 40× oil immersion objective lens (NA = 1.30) for an overview intensity quantification. Three fields of 320 × 320 *μ*m (2048 × 2048) for each sample were captured as a z-stack of 30–40 images with a z-step of 0.32* μ*m. Image projection and intensity quantification for HS and HA of glycocalyx were performed by Zeiss ZEN and NIH ImageJ.^24^ For quantifying the GCX (HS and HA) thickness, we used a lens with higher magnification and numerical aperture (63×/NA1.4) to scan fields of 160 × 160* μ*m (2048 × 2048) and a z-stack of 50–60 images with a z-step of 0.2 *μ*m. The thickness of the GCX was estimated by the full width at half maximum (FWHM) as described in Appendix C.

### Imaging HS and HA of glycocalyx by STORM

**FIG. 5.**
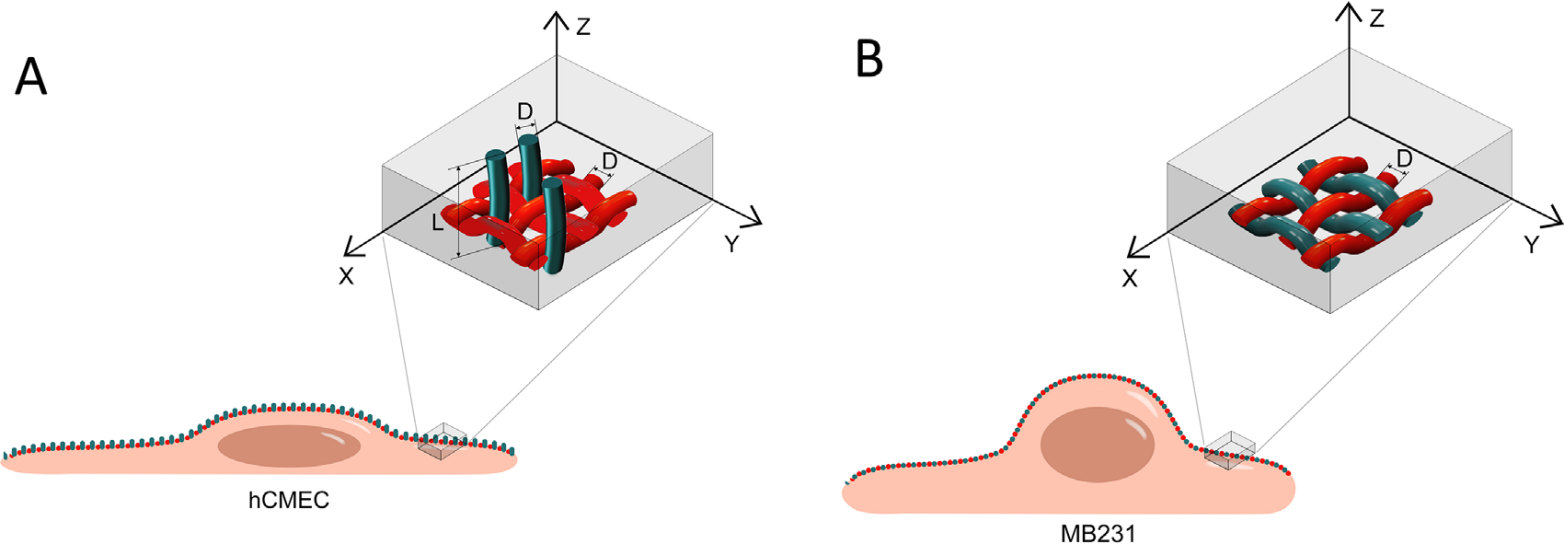
Image acquisition locations for HS and HA elements on hCMEC and MB231. (a) On hCMEC and (b) on MB231. The horizontal x-y plane (lateral plane) is focused on the cell apical surface, and the vertical direction is z (axial direction) with its origin at the cell surface. The movie of the raw images (blinking dots) for the ROI (40 × 40 *μ*m) was collected from the cell surface up to ∼1.5 *μ*m from the surface. The enlarged insets show the views of the hypothetical organization of HS elements (green) and HA elements (red) for these two types of cells. D indicates the diameter, and L indicates the length.

**FIG. 7.**
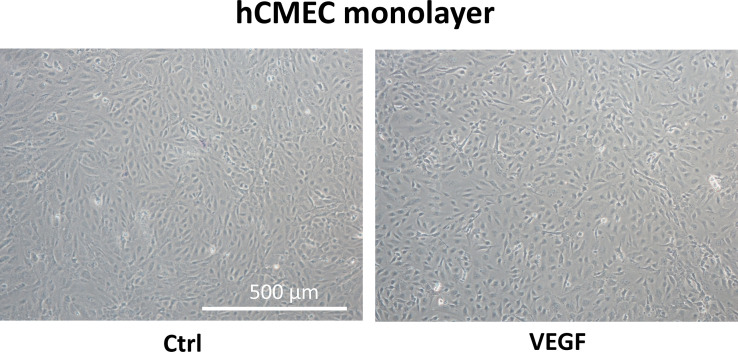
Phase contrast images of hCMEC monolayer. Left is the image for the control and right for that after 2 h treatment with 1 nM VEGF.

**FIG. 8.**
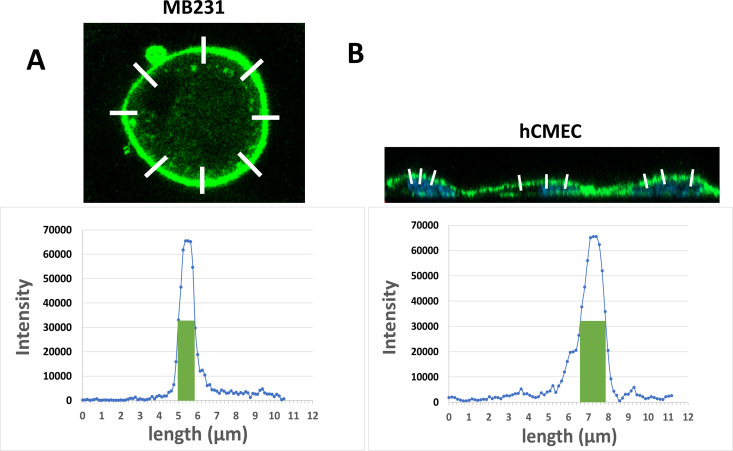
Schematic for quantification of glycocalyx thickness from the confocal images. (a) Estimating the thickness of glycocalyx (HS or HA) on a MB231 cell. A white line was drawn perpendicular to the cell surface (upper figure) and the intensity profile along the line was plotted (bottom figure). The thickness of the glycocalyx at that location was estimated as the full width at half maximum (FWHM) (the width of the green bar). The averaged thickness from 8 locations along the circumference of a cell (8 white lines) was taken as the glycocalyx thickness of that MB231 cell. (b) Estimating the thickness of glycocalyx (HS or HA) on a hCMEC. The averaged FWHM along 3 white lines perpendicular to the cell surface was taken as the glycocalyx thickness of that hCMEC.

The method for imaging HS and HA of GCX by STORM is the same as in our previous study.^33^ The N-STORM system (Nikon Instruments INC., Melville, NY) with a 100×/1.49 oil immersion lens was used for imaging. The 405 nm wavelength laser activated the fluorescent reporters of Alexa Fluor 647 to generate the 3D images of HS and HA of GCX at cell surface (see Appendix A for the working principle of STORM). Three fields of 40 × 40 *μ*m (256 × 256) on the cell apical surface of each sample were obtained based on 40,000 of EM-CCD captured images at a capturing speed of 19 ms/frame. Figure 5 demonstrates the location of imaging on the surface of hCMEC and MB231. It took 15–20 min to obtain the image movie of one field (40 *μ*m x 40 *μ*m) with a data size of 1.5–3 GB. The raw image movies of blinking dots were processed by the analyzing software in the N-STORM system to generate the 3D images shown in Figs. 2 and 3.

### Data analysis

For all the data analyzed from either STORM or confocal images, data were presented as means ± SD (standard deviation). Statistical analysis was performed by a student T-test in Microsoft Excel. For each cell type, all the data were from the images collected from the samples out of three biological replicates for the control and VEGF treatment. The statistics from the three biological replicates was also performed, and the conclusions are the same as those by the statistics from all the data.

#### Confocal image analysis

For quantifying intensity of HS and HA at hCMEC monolayer, the averaged intensity of three fields (each field 320 × 320 *μ*m) collected by 40×/NA1.3 lens was measured for each sample. Three samples were measured for each case. The average of 3 control samples was used for the normalization in HS and HA quantification. For quantifying intensity of HS and HA at MB231, three samples with 10 cells in each sample were analyzed for each case for the HS and HA at MB231. The average of 3 control samples was used for the normalization in HS and HA quantification. For quantifying the thickness of HS and HA, three samples with 10 cells in each sample were analyzed for each case for both hCMEC and MB231.

#### STORM image analysis

**FIG. 9.**
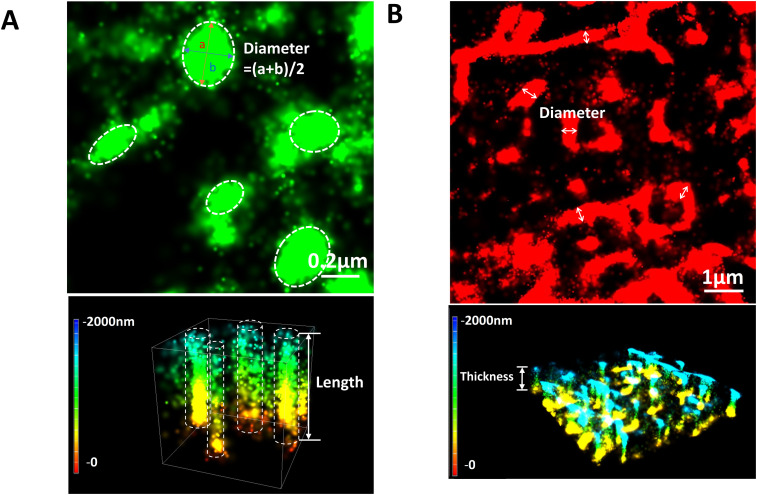
Quantification of glycocalyx ultrastructural parameters from STORM images. (a) Upper figure shows the top view of HS elements at hCMEC. The scale bar is 0.2 *μ*m. The white dashed line circled regions indicate several HS elements. The equivalent diameter of a HS element is estimated as the average of the lengths for the long axis (a) and short axis (b). The diameter of the HS element with “a” and “b” labeled is (31 + 240 nm)/2 = 277 nm. Bottom figure shows the 3D view of HS elements. The length is measured from the cell surface to the tip using the NIS-Elements software in the N-STORM system. The color bar is the length scale, where the cell surface is at the origin (0). The white dashed line formed columns represent several HS elements. The one with the Length labeled is 1230 nm long. (b) Upper figure shows the top view of HA elements at hCMEC. The scale bar is 1 *μ*m. The diameter of a HA element is estimated as the distance between edges of a medium sized segment in that element (indicated by the line with double arrow heads). The diameter of the HA element labeled with “diameter” is 421 nm. Bottom figure shows the 3D view of HA elements, which appear to form a network covering the cell surface. The thickness of the cover is estimated by the averaged distance between three locations from the bottom to the top for a region of 1–2 × 1–2 *μ*m (12.5%–25% of the size of the region shown in B), which is measured by the NIS-Elements software. Thickness from 30 regions of 1–2 × 1–2 *μ*m is measured for each case. The location with the label “thickness” is 455 nm thick. The diameters of HS and HA of MB231 as well as the corresponding thicknesses are determined by the same method for the HA of hCMEC.

For analyzing data movies collected from the STORM, the single-emitter centroid algorithm was applied to estimate the 3D locations of activated fluorophores in the data movie and produced a 3D STORM image with the spatial resolutions of ∼20 nm in the lateral plane and ∼50 nm in the axial direction, respectively. This algorithm is included in the software installed in the N-STORM system. Ultrastructure parameters of the glycocalyx elements were then estimated based on 3D STORM images.^33^ For the HS element perpendicular to the hCMEC surface [e.g., sixth row in Fig. 2(a)], we assumed that it is a circular cylinder with the equivalent diameter averaging the lengths of long and shot axes from the elliptic-like cross section. The length of HS at hCMEC, the diameter of HA at hCMEC, and those of HS and HA at MB231 are defined in Fig. 5. We used the NIS-Elements software in the N-STORM system to estimate the length of HS at hCMEC individually from the 3D images [e.g., sixth row in Fig. 2(a)] and the diameter of HA at hCMEC and those of HS and HA at MB231 from the 2D images of these elements shown in the third row of Figs. 2(b), 3(a), and 3(b). Appendix D (Fig. 9) demonstrates how to determine the diameter, length, and thickness of HS and HA elements from the STORM images.

For the length of HS at hCMEC, n = 100–110 elements, each was measured for the control and VEGF treatment. For the diameter of HA at hCMEC and the diameters of HS and HA at MB231, n = 100–200 elements were measured for each case. For the thickness of HA at hCMEC, and those of HS and HA at MB231, n = 30 regions (1–2 × 1–2 *μ*m for each region) were measured. For each 1–2 × 1–2 *μ*m (x-y) region, 3D image was reconstructed by the STORM software and the thickness of that region was averaged from 3 locations from the x-z view by the NIS-Elements software in the N-STORM system. For the coverage of HA at hCMEC and HS and HA at MB231, n = 30–50 regions (1–2 × 1–2 *μ*m for each region) were analyzed for each case. The coverage is the percentage of the surface covered by these elements. The coverage of HS at hCMEC was calculated by converting the density (number of elements/*μ*m^2^) using density x π(D/2)^2^, where D is the average diameter of the HS element at hCMEC.

## Figures and Tables

**FIG. 6.**
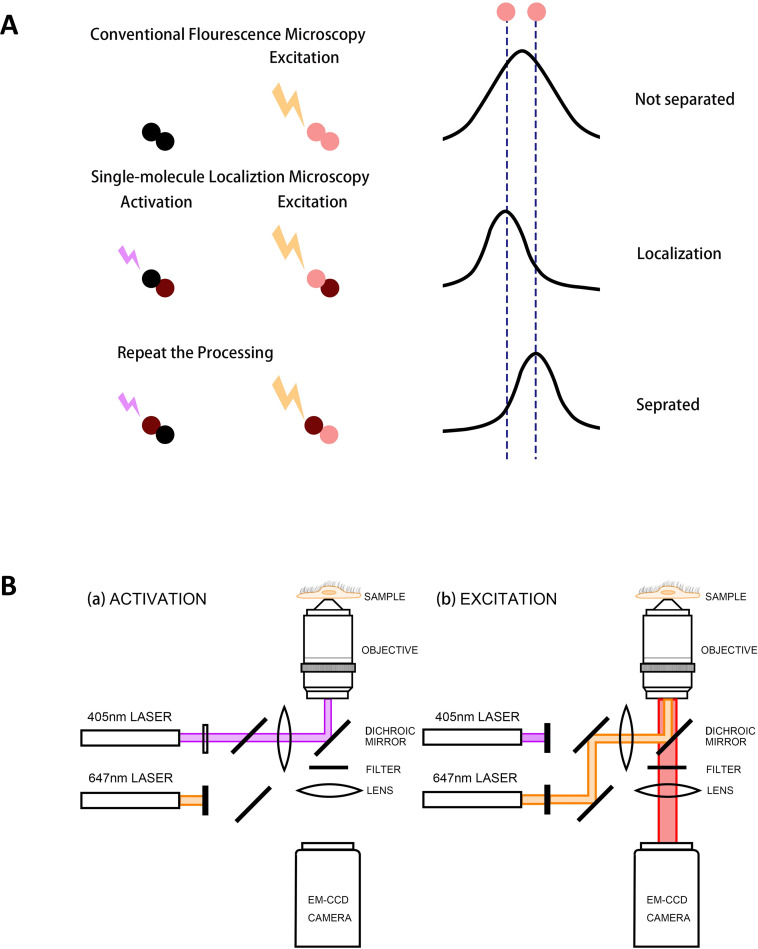
Illustration for the working principle and the optical setup of STORM. (a) The top row demonstrates that the two fluorophores (located along the two dashed lines) activated and excited simultaneously by conventional microscopy. Due to the diffraction limit, the two fluorophores cannot be separated, resulting in a blurring image. The middle and bottom rows show the principle of STORM for the localization of a single molecule to a nanometer accuracy. The fluorophores are activated and excited not simultaneously, but sequentially to be localized and separated with each other. This technique can overcome the diffraction barrier for the conventional fluorescence microscopy. (b) A representative optical setup of STORM for the fluorophore AF 647. (a) The optical path in the activation process. The 405 nm wavelength laser activates the photo-switchable fluorophore AF 647, enabling the excitation. (b) The optical path in excitation and imaging processes. The 647 nm laser excites the activated “on” fluorophore AF 647, and its emission is recorded by a camera. Adapted with permission from Y. Xia and B. M. Fu, “Investigation of endothelial surface glycocalyx components and ultrastructure by single molecule localization microscopy: Stochastic optical reconstruction microscopy (STORM),” Yale J. Biol. Med. **91**(3), 257–266 (2018). Copyright 2018 Authors, licensed under a Creative Commons Attribution (CC BY) license.

## Data Availability

The data that support the findings of this study are available from the corresponding author upon reasonable request.

## APPENDIX A: WORKING PRINCIPLE OF STORM

Before the super-resolution microscopy was invented, the spatial resolution of a fluorescence optical microscope was limited by the diffraction, due to the wave nature of light. The diffraction of a single fluorescence molecule can be described as the point spread function (PSF). When the light of wavelength λ excites the fluorophore (emitter), the intensity profile of the spot is defined as the PSF with the width (or diameter) ∼0.6 λ/NA; NA is the numerical aperture of the objective. As a result, two identical emitters separated by a distance less than the width of the PSF would appear as a single object, not distinguished from each other [top plot at the right panel in Fig. 6(a)]. The diffraction-limited image resolution, for a high numerical aperture objective lens (e.g., 1.3–1.4) and 500–700 nm wavelength light, is ∼200–300 nm in the lateral direction and ∼500–600 nm in the axial direction, for a conventional fluorescence microscope.^31^ The diffraction limit led the development of various microscopy, either to decrease the wavelength out of the range of visible lights, such as electron microscopy, or to get rid of the lights, such as scanning probe microscopy. Until 1993, Betzig and Chichester developed a single-molecule localization method to achieve nanometer scale resolution for a fluorescence optical microscope.^63^ Because of this revolutionary work, Betzig received the 2014 Nobel Prize in Chemistry for “allowing biologists to study subcellular activity in finer detail than ever before.”

The main concept of the single-molecule localization microscopy is to light the molecule, in turn, to achieve the nanometer-level accuracy of their position and reconstruction into a super-resolution image, such as Stochastic Optical Reconstruction Microscopy (STORM) or photoactivated localization microscopy (PALM). Figure 6(a) illustrates the working principle of STORM by employing photo-switching mechanisms to stochastically activate individual molecules (photo-switchable or photoactivatable fluorophores) within the diffraction-limited region at different times. Then, images with sub-diffraction limit resolution are reconstructed from the measured positions of individual fluorophores. To localize a molecule, the emitter at that molecule should not have any overlap with its neighbors, so that it can be isolated and fitted into the PSF [middle and bottom plots in Fig. 6(a)]. One approach to let the fluorescence molecule emitting in turn is to make the fluorophore blink. To achieve the photo-blinking of the fluorophore is to switch between light and dark states, which are usually called “on” and “off.” On is the state in which the fluorophore can be excited, and its emission can be detected by the camera. The off state is in which the fluorophore cannot be excited by the laser, including the temporarily blinking or permanently bleaching. The most important factor for STORM to accomplish sub-diffraction resolution is that the photo-switchable fluorophores are used to keep neighboring molecules in different states, on or off, enabling to be distinguished from each other.^64^ A representative optical setup of STORM for the fluorophore Alexa Fluor 647 (AF 647) is shown in Fig. 6(b). From one optical path, the 405 nm wavelength laser activates the photo-switchable fluorophore AF 647, enabling the excitation. Then, through another optical path, the 647 nm laser excites the activated on fluorophore and its emission is recorded by a camera.

The number of emitters and the image collecting time are the determinants of the efficiency and accuracy of any microscopy. The higher population of the emitter would have a higher image collection efficiency; however, the distance between neighboring emitters must be greater than their PSF to distinguish each other for conventional microscopy. To trade the superspatial resolution (accuracy), STORM sacrifices its temporal resolution (efficiency) by switching the state and sequentially exciting the emitters at high density. Rust *et al.* employed organic dyes and fluorescent proteins as photo-switchable emitters to trade temporal resolution for a superspatial resolution (∼20 nm lateral and ∼50 nm axial at present, can go down to a couple of nanometers if using smaller peptides or anti-body fragments instead of currently used whole anti-bodies), which is an order of magnitude higher than conventional confocal microscopy. STORM can perform 2D, 3D,^31^ and multicolor^65^ imaging. In each frame of a STORM movie, a set of emitters is randomly activated so that locations of single emitters isolated within their PSFs can be spatially resolved.

Since it was developed, STORM has been employed by many researchers because of its ability to obtain the spatio-chemical organization and structure at nanometer scales. Several subcellular structures, including microtubules, actin, clathrin-coated pits, mitochondria, endoplasmic reticulum, and focal adhesion complexes, have been visualized by STORM,^32^ as well as protein organization in bacteria,^66^ molecular architecture of nerve synapses,^67^ and surface glycocalyx components and ultrastructure on endothelial cells.^33^

## APPENDIX B: PHASE CONTRAST IMAGES OF hCMEC Monolayer

Figure 7 demonstrates the phase contrast images of hCMEC monolayer under control and after VEGF treatment.

## APPENDIX C: QUANTIFICATION OF GLYCOCALYX THICKNESS FROM THE CONFOCAL IMAGES

Figure 8 shows how to quantify the glycocalyx thickness from the confocal images.

## APPENDIX D: QUANTIFICATION OF DIAMETER, LENGTH, AND THICKNESS OF HS AND HA ELEMENTS FROM THE STORM IMAGES

Figure 9 shows how to quantify the diameter, length, and thickness of HS and HA elements from the STORM images.
